# Circulating Fetuin‐A concentrations in rheumatic diseases: a systematic review and meta‐analysis

**DOI:** 10.1111/eci.14365

**Published:** 2024-11-28

**Authors:** Biagio Di Lorenzo, Stefano Zoroddu, Arduino A. Mangoni, Panagiotis Paliogiannis, Gian Luca Erre, Ciriaco Carru, Angelo Zinellu

**Affiliations:** ^1^ Department of Biomedical Sciences University of Sassari Sassari Italy; ^2^ Department of Medicine and Surgery LUM University Casamassima Italy; ^3^ Discipline of Clinical Pharmacology, College of Medicine and Public Health Flinders University Bedford Park South Australia Australia; ^4^ Department of Clinical Pharmacology, Flinders Medical Centre Southern Adelaide Local Health Network Bedford Park South Australia Australia; ^5^ Department of Medicine, Surgery and Pharmacy University of Sassari Sassari Italy; ^6^ Anatomic Pathology and Histology Unit University Hospital (AOU) of Sassari Sassari Italy; ^7^ Rheumatology Unit University Hospital (AOU) of Sassari Sassari Italy; ^8^ Medical Oncology Unit University Hospital (AOU) of Sassari Sassari Italy

**Keywords:** biomarker, Fetuin‐A, rheumatic diseases, α2‐Heremans‐Schmid glycoprotein

## Abstract

**Background:**

Rheumatic diseases (RDs) include a broad group of disabling conditions with different phenotypes, from autoimmune to autoinflammatory, degenerative, metabolic or mixed manifestations. With the continuous efforts to identify therapeutic targets for new biologic drugs to treat overt clinical manifestations, research is also focusing on the discovery of new biomarkers to diagnose and manage early disease stages. In this context, we conducted a systematic review and meta‐analysis of Fetuin‐A (FtA), a glycoprotein synthesized by the liver that participates in several biological processes and has been proposed as a biomarker for several disorders, including rheumatoid arthritis.

**Methods:**

A systematic search in PubMed, Scopus and Web of Science, from inception to the 24th of August 2024, led to the identification of 13 manuscripts from 219 records; six additional studies were identified through reference hand‐search, for a total of 19 studies.

**Results:**

There was a significant decrease in FtA concentrations in RD patients (standardized mean difference, SMD = −.91; 95% CI −1.43 to −.39, *p* = .001), with no substantial contribution from any individual study nor publication bias. The effect size was significantly associated with erythrocyte sedimentation rate, various lipid fractions, geographical area of study conduction, study design and specific type of RD.

**Conclusion:**

In conclusion, our study identified significant reductions in FtA concentrations in RD patients versus healthy controls. These alterations were significantly associated with specific study and patient characteristics. Further research is required to identify the exact pathophysiological mechanisms underlying these alterations and the possible utility of measuring FtA for the diagnosis and management of RDs.

## Graphical abstract

In this systematic review with meta‐analysis, we identified 19 eligible studies to determine whether there is any change in Fetuin‐A (FtA) concentrations in patients with rheumatic diseases. We observed a significant decrease in FtA concentrations in RD patients (standardized mean difference, SMD = −.91; 95% CI −1.43 to −.39, *p* = .001). Moreover, the effect size was significantly associated with erythrocyte sedimentation rate, various lipid fractions, geographical area of study, study design and specific types of rheumatic diseases.

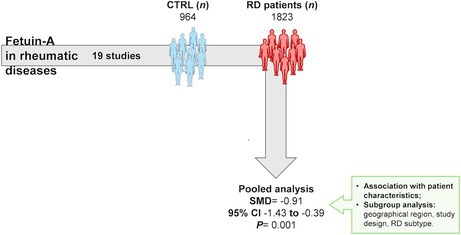

## 1 INTRODUCTION

Rheumatic diseases (RDs) represent a group of different disabling conditions affecting between 9.8% and 33.2% of the general population,^1^ albeit with significant geographic variation in the estimated incidence and prevalence. A relatively high prevalence of RDs has been reported in industrialized nations and urban areas and in females.^2^, ^3^ RDs refer to a broad category of disorders that typically affect the musculoskeletal system and many different tissues and are commonly categorized in autoimmune, autoinflammatory, degenerative and metabolic disorders.^4^ Autoimmune RDs are characterized by immune response dysregulation that triggers immune cell activation against autoantigens, causing unwarranted inflammation and tissue damage. This group encompasses highly prevalent disorders, such as rheumatoid arthritis (RA), systemic lupus erythematosus (SLE) and systemic sclerosis (SSc).^5^ A dysregulation of the innate immune response is also common in autoinflammatory RDs. However, this class of RDs differs from the former for the generation of inflammation in the absence of antigen‐specific T cells or high‐titre auto‐antibodies.^6^ Finally, degenerative RDs, for example, osteoarthritis (OA), lead to a progressive loss of synovial joint function as a result of articular cartilage degradation,^7^ while metabolic RDs are the result of alterations in bone mineral metabolism, causing a decrease in bone mineral density. Osteoporosis and gout are the most common disorders of this group.^4^ While significant progress has been made in the diagnosis and treatment of clinically overt RDs, particularly with the introduction of new biologic drugs,^8^, ^9^ research on identifying new biomarkers is still ongoing.^10^, ^11^ Pending the results of these studies, conventional inflammatory biomarkers such as C‐reactive protein (CPR) and erythrocyte sedimentation rate (ESR) are used in conjunction with clinical evaluation for early disease diagnosis.^12^, ^13^

Fetuin‐A (FtA) is a glycoprotein synthesized by the liver and once released in the bloodstream participates into several biological processes, including insulin signalling, calcium and bone metabolism control and inflammation.^14^ Additionally, FtA functions as an adipogenic and atherogenic factor, protease inhibitor and plasma carrier protein for phosphate and calcium, controlling their concentrations and preventing ectopic calcification.^15^, ^16^ FtA serum concentrations can be measured with commercially available enzyme‐linked immunosorbent assay (ELISA) kits and can be influenced by several factors, including age, comorbidities, body mass index (BMI), as well as individual kits as a result of variability in post‐translational modifications.^17^, ^18^ However, particular importance should be given to post‐Since FtA has been already proposed as biomarker for several disorders, including rheumatoid arthritis, depression, coronary artery disease, metabolic syndrome, aortic valve stenosis and type 2 diabetes,^14^, ^19^ the aim of our study was to critically appraise the available evidence regarding the concentrations of FtA in patients with RDs and healthy subjects.

## 2 MATERIALS AND METHODS

### 2.1 Search strategy, eligibility criteria and study selection

The following terms were used for identifying relevant articles in PubMed, Web of Science and Scopus from inception until the 24th of August 2024: ‘Fetuin A’ OR ‘α2‐Heremans‐Schmid glycoprotein’ AND ‘rheumatic diseases’ OR ‘rheumatoid arthritis’ OR ‘psoriatic arthritis’ OR ‘reactive arthritis’ OR ‘ankylosing spondylitis’ OR ‘systemic lupus erythematosus’ OR ‘systemic sclerosis’ OR ‘scleroderma’ OR ‘Sjogren's syndrome’ OR ‘connective tissue diseases’ OR ‘vasculitis’ OR ‘Behçet's disease’ OR ‘idiopathic inflammatory myositis’ OR ‘polymyositis’ OR ‘dermatomyositis’ OR ‘gout’ OR ‘pseudogout’ OR ‘systemic vasculitis’ OR ‘ANCA‐associated vasculitis’ OR ‘takayasu arteritis’ OR ‘polyarteritis nodosa’ OR ‘osteoarthritis’ OR ‘fibromyalgia’ OR ‘granulomatous polyangiitis’ OR ‘henoch‐schonlein purpura’ OR ‘wegener granulomatosis’. The complete search strategy is available as Appendix S1.

The selection of relevant publications, the systematic review and meta‐analysis were performed according to the Preferred Reporting Items for Systematic reviews and Meta‐Analyses (PRISMA) 2020 statement,^20^ selecting case–control studies that included FtA measurements in RD and healthy individuals. Paediatric cohorts (under 18), studies that were not written in English, case report and case series (*n* ≤ 10) were excluded. Study related variables, VEGF concentrations and clinical and demographical information have been collected into an electronic database. The processes of study selection and data extraction were conducted by two independent investigators (BDL and SZ); any discrepancy was resolved by a third investigator (AZ).

The Joanna Briggs Institute (JBI) Critical Appraisal Checklist for analytical studies was used to assess the risk of bias^21^ while the certainty of evidence was assessed using the Grades of Recommendation, Assessment, Development and Evaluation (GRADE) Working Group system.^22^ The study complied with the PRISMA 2020 statement^20^ and the study protocol was registered in the International Prospective Register of Systematic Reviews (PROSPERO, CRD42024526858).

### 2.2 Statistical analysis

Medians and interquartile or min‐max ranges were converted into means and standard deviations.^23^ To avoid inconsistencies among studies due to the use of different ELISA kits, differences in FtA concentrations were summarized in forest plots of standardized mean differences (SMD) and 95% confidence intervals (CIs), with values of *p* < .05 considered statistically significant. SMD values were categorized into small (SMD < .5), moderate (SMD: .5–.8); and large (SMD >.8).^24^

The Q and the *I* ^2^ statistics were used to assess the inconsistency and the heterogeneity of SMD.^25^, ^26^ The impact of each individual study on the overall effect size was assessed through the sensitivity analyses,^27^ while the publication bias and its correction were tested with the Begg's, the Egger's tests and the Duval and Tweedie ‘trim and fill’ procedure, respectively.^28^, ^29^, ^30^

The association of the effect size with clinical and demographical variables was evaluated in the univariate meta‐regression and subgroup analyses. Statistical analyses were performed using Stata 14 (STATA Corp., College Station, TX, USA).

## 3 RESULTS

### 3.1 Systematic research

**FIGURE 1:**
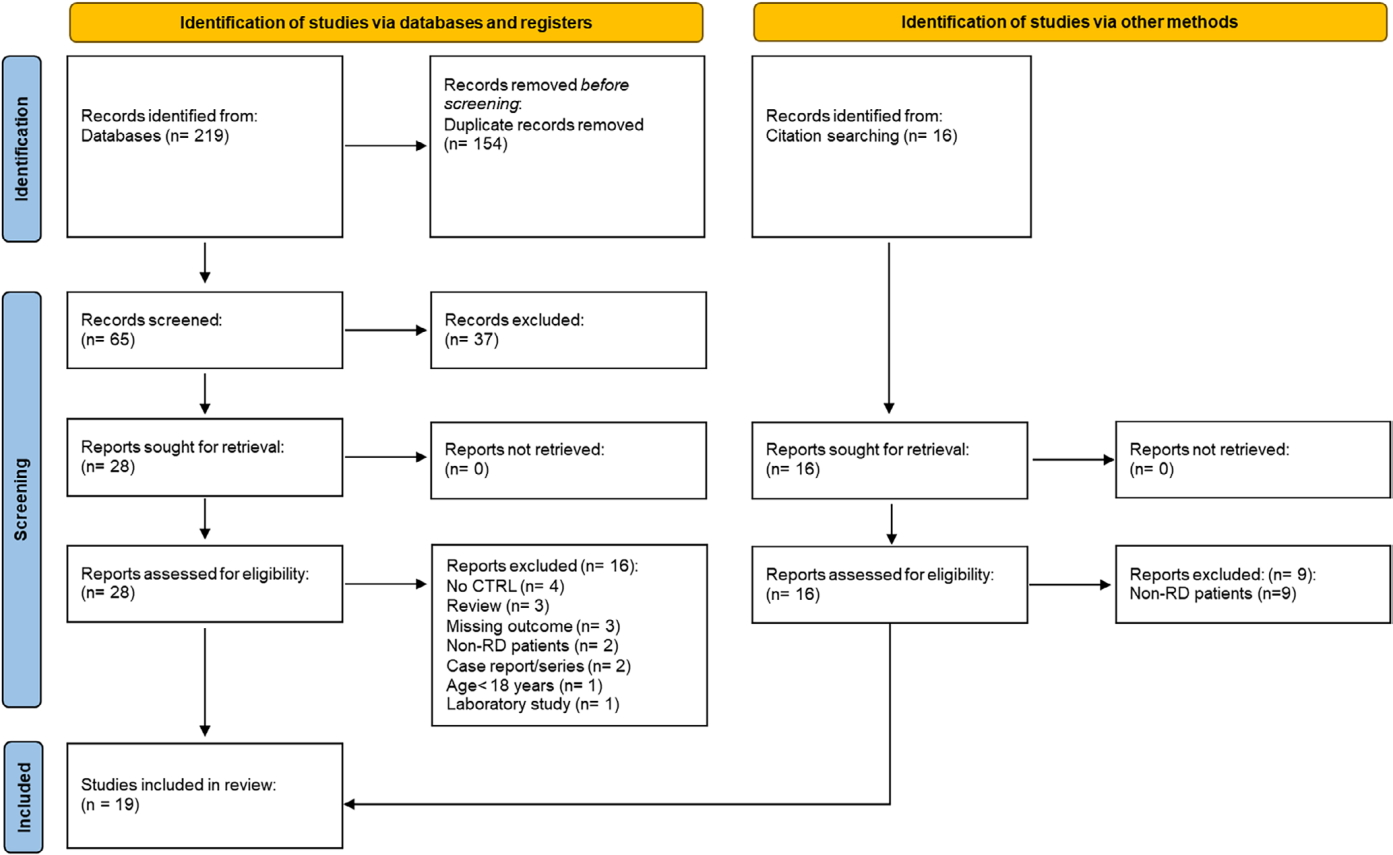
PRISMA 2020 flow diagram for systematic reviews.

The result of the systematic search consisted of 219 records: 154 were duplicates while 65 unique. After screening the title and abstract for relevance, the full text of 28 manuscripts was assessed. This, in turn, led to the inclusion of 13 manuscripts and the exclusion of 15 for not reporting the required outcome (*n* = 4), not having a control cohort (*n* = 4), being a review (*n* = 3), case report or series (*n* = 2), animal study (*n* = 1) or for including a paediatric cohort (*n* = 1). Six additional records were identified by hand searching of reference lists, leading to the inclusion of 19 studies in our systematic review and meta‐analysis (Figure 1).^31^, ^32^, ^33^, ^34^, ^35^, ^36^, ^37^, ^38^, ^39^, ^40^, ^41^, ^42^, ^43^, ^44^, ^45^, ^46^, ^47^, ^48^, ^49^

The risk of bias was low in 16 studies and moderate in the remaining three (Table S1). The initial level of certainty was ranked as low given the cross‐sectional design of the selected studies.

### 3.2 FtA in rheumatic diseases

#### 3.2.1 Study characteristics

We identified 19 original studies including 24 group comparators, with 1823 RD patients (mean age 47.8 years, 61.2% females) and 964 healthy controls (mean age 41.7 years, 49.4% females, Table 1).^31^, ^32^, ^33^, ^34^, ^35^, ^36^, ^37^, ^38^, ^39^, ^40^, ^41^, ^42^, ^43^, ^44^, ^45^, ^46^, ^47^, ^48^, ^49^ The studies were conducted in the following countries: eight in Turkey,^32^, ^37^, ^38^, ^40^, ^42^, ^43^, ^44^, ^48^ four in Egypt,^33^, ^34^, ^35^, ^39^ two in Poland^41^, ^49^ and one each in China, India, Iraq, Japan, Russia.^31^, ^36^, ^45^, ^46^, ^47^ Sixteen were conducted retrospectively,^31^, ^32^, ^33^, ^34^, ^35^, ^36^, ^37^, ^38^, ^39^, ^41^, ^42^, ^43^, ^45^, ^47^, ^48^, ^49^ and the remaining three prospectively.^40^, ^44^, ^46^ The complete list of laboratory parameters and clinical and demographical characteristics of the two study groups is described in the Tables S2 and S3.

**TABLE 1:** Summary of study characteristics.

Study name, Year	Country	Study design	RD patients			CTRL	
			Disease	*n*	FtA (ug/ml)	*n*	FtA (ug/ml)
					Mean ± SD		Mean ± SD
Sato H et al., 2007	Japan	R	RA	102	249.8 ± 84.1	155	425 ± 93
Sari I et al., 2010	Turkey	R	AS	45	1023.5 ± 171.6	29	856.9 ± 207.9
Mosa FO et al., 2012	Egypt	R	SLE	100	500 ± 13	50	700 ± 70
Abdel‐Wahab AF et al., 2013	Egypt	R	SLE	40	505 ± 100	20	780 ± 200
Keshk WA et al., 2013	Egypt	R	SLE	30	.000000515 ± .00000016	15	.00000095 ± .0000001
Xiao J et al., 2013	China	R	OA	215	364.2 ± 84.4	76	525.5 ± 90
Tuylu T et al., 2014	Turkey	R	AS	94	1102.7 ± 109.1	68	1080 ± 130
Gökmen et al., 2015	Turkey	R	AS	47	.98 ± .2	30	1.16 ± .22
Shafik NM et al., 2015	Egypt	R	RA	40	.000252 ± .000021	20	.000447 ± .000028
Uyar B et al., 2015	Turkey	P	BD	26	.0073 ± .0054	25	.0037 ± .0066
Przepiera‐Będzak H et al., 2016	Poland	R	SpA	191	606 ± 155.3	30	709.1 ± 169.3
	Poland	R	AS	81	601 ± 147.8	30	709.1 ± 169.3
	Poland	R	PA	76	599.5 ± 152.7	30	709.1 ± 169.3
	Poland	R	SAPHO	34	632.2 ± 179	30	709.1 ± 169.3
Tekeoğlu İ et al., 2016	Turkey	R	RA	60	926 ± 302	20	1052 ± 299
Harman H et al., 2017	Turkey	R	SpA	55	793 ± 411.6	28	418 ± 97
	Turkey	R	RA	38	687 ± 437	28	418 ± 97
Sag S et al., 2017	Turkey	P	BD	58	1256 ± 223	20	1052 ± 299
Papichev EV et al., 2019	Russia	R	RA	110	765.7 ± 120.7	30	812.9 ± 76.2
Kumar PA et al., 2021	India	P	AS	60	.092 ± .017	60	.14 ± .033
Ibrahim S et al., 2022	Iraq	R	RA	100	.00047 ± .00032	100	.000278 ± .000177
Karadeniz H et al., 2022	Turkey	R	TA	32	.076 ± .06	20	.094 ± .071
	Turkey	R	GP	28	.071 ± .07	20	.094 ± .071
Przepiera‐Będzak H et al., 2022	Poland	R	SpA	161	599.1 ± 149.3	30	709.1 ± 169.3

*Note*: The study design (P, prospective; R, retrospective), disease (AS, axial spondylarthritis; BD, Behcet's disease; GP, granulomatous polyangiitis; OA, osteoarthritis; PA, psoriatic arthritis; RA, rheumatoid arthritis; SAPHO, synovitis‐acne‐pustulosis‐hyperostosis‐osteitis syndrome; SLE, systemic lupus erythematosus; SpA, spondylarthritis; TA, Takayasu arteritis), sample size and FtA levels (ug/ml) as mean ± SD.

#### 3.2.2 Results of individual studies and syntheses

**FIGURE 2:**
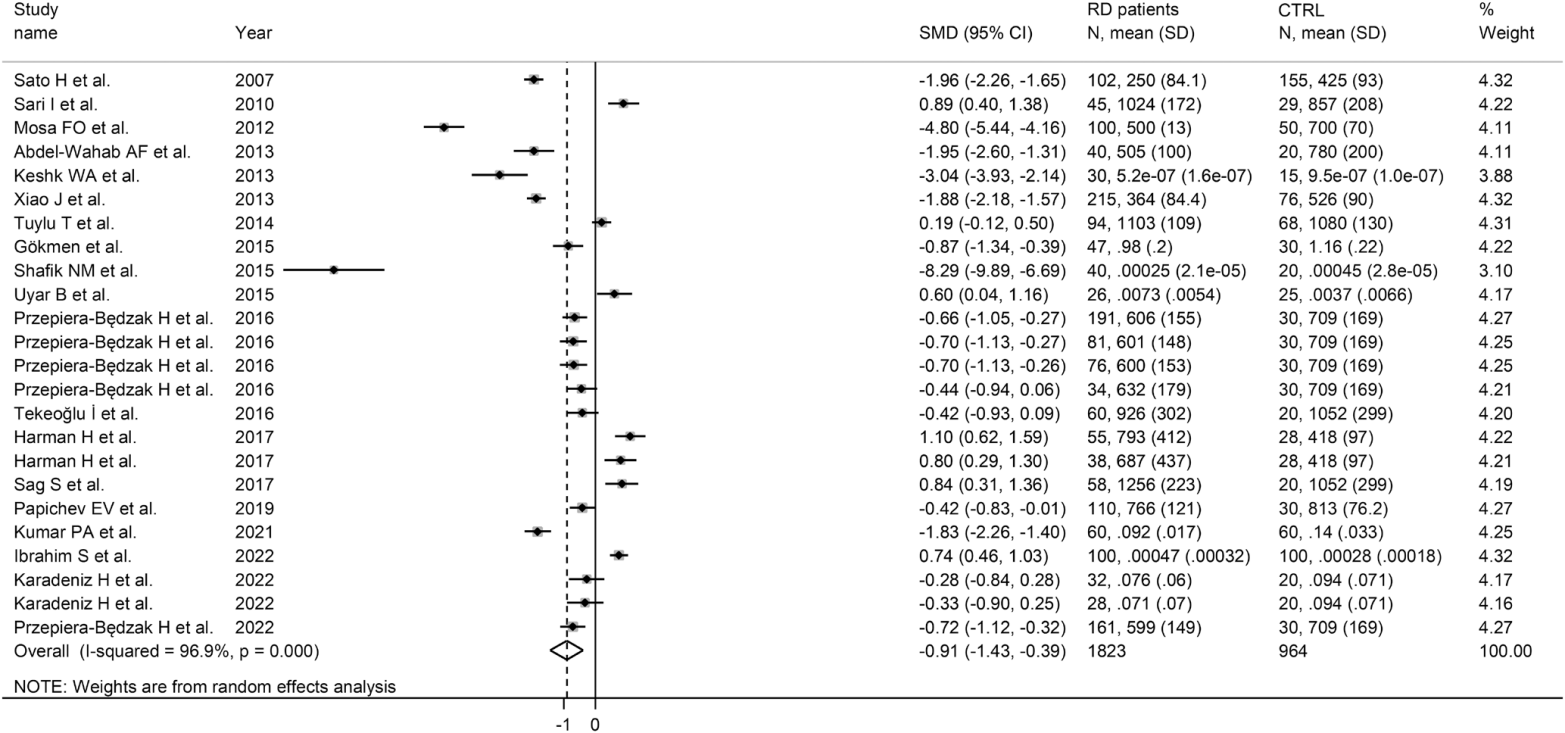
Synthesis forest plot of FtA concentrations in RD patients and healthy individuals.

**FIGURE 3:**
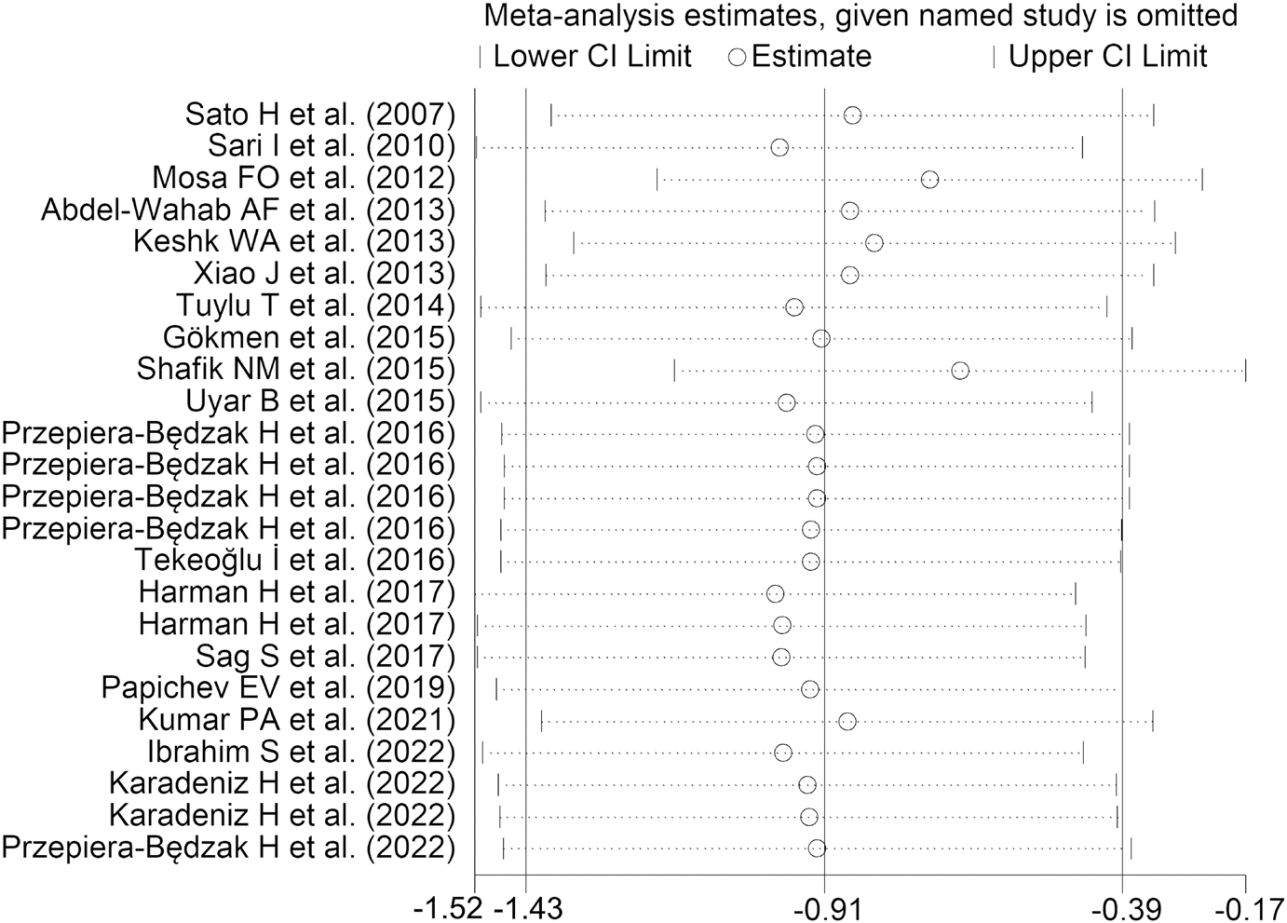
Sensitivity analysis of individual study impact on the observed effect size.

The synthesis of the selected studies indicated a statistically significant reduction in FtA concentrations in subjects with RDs when compared to controls, with an observed large pooled SMD = −.91 (95% CI −1.43 to −.39, *p* = .001), and extreme heterogeneity across studies (*I* ^2^ = 96.9%, *p* < .001, Figure 2). Consequently, the random effects model was applied. Overall, no single studies substantially affected the observed effect size, as demonstrated by the sensitivity analysis (Figure 3): the SMD ranged between −1.00 and −.67 when each individual study was sequentially excluded from the pooled analysis. Also, no publication bias emerged from the Begg's and Egger's test (*p* = .64 and *p* = .25, respectively) and, therefore, no correction was applied by the ‘trim‐and‐fill’ procedure (Figure S1).

#### 3.2.3 Meta‐regression and subgroup and analysis

**FIGURE 4:**
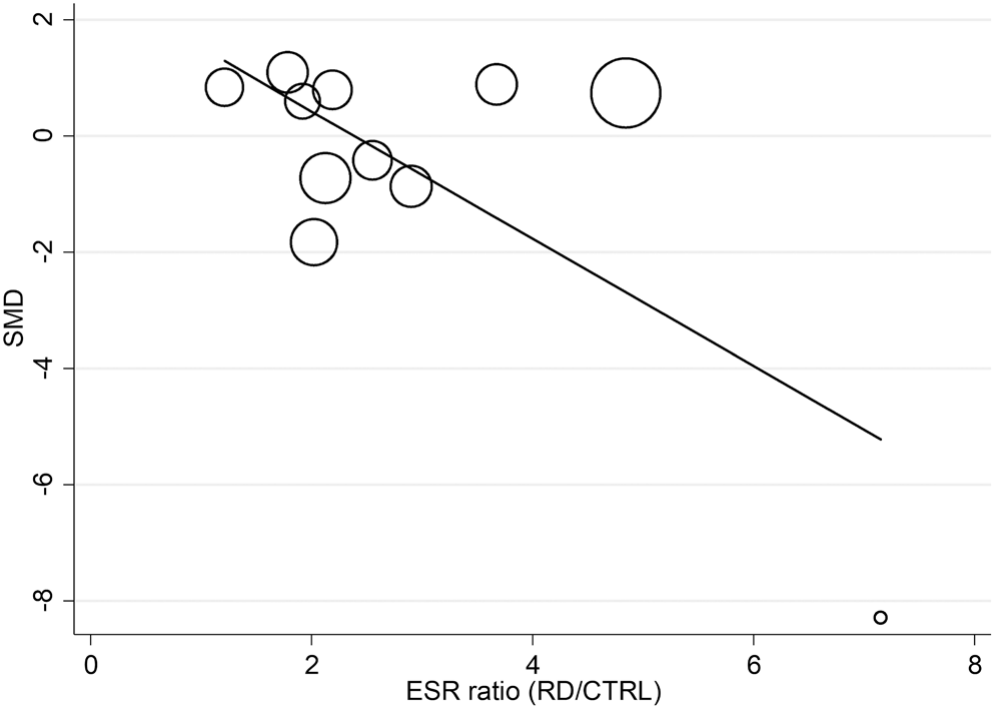
Bubble plot reporting univariate meta‐regression analysis between the effect size and ESR.

**FIGURE 5:**
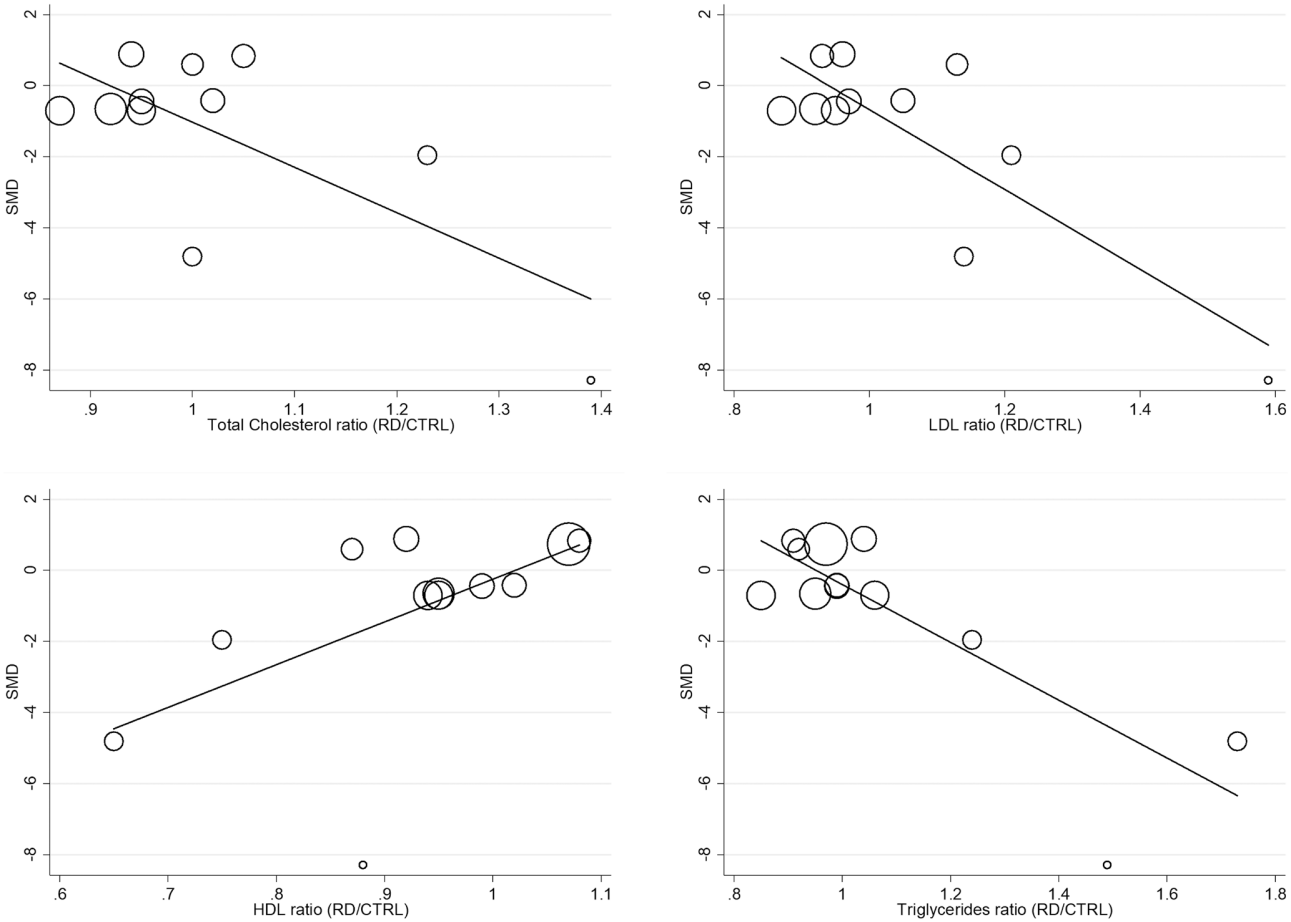
Bubble plots reporting univariate meta‐regression analysis between the effect size and lipid fractions (total cholesterol, HDL, LDL and triglycerides).

Univariate meta‐regression analysis was performed to assess if the between‐group differences in FtA concentrations were associated with predefined study and patient characteristics. No significant associations between the FtA effect size and the year of publication (t = 1.36; *p* = .19); median RD duration (t = .54; *p* = .60); age (t = .87; *p* = .40); sex (t = −.26; *p* = .80); body mass index (t = 1.68; *p* = .13); CRP (t = .67; *p* = .52); or use of nonsteroidal anti‐inflammatory drugs (t = −.86; *p* = .42). By contrast, other variables were significantly associated, for example, ESR (t = −3.00; *p* = .015) and lipid fractions (total cholesterol t = −2.95, *p* = .016; LDL t = −4.36, *p* = .002; HDL t = 2.20, *p* = .053; and triglycerides t = −4.82, *p* = .001, Figures 4 and 5). Subgroup analysis of the pooled SMD was conducted on the following variables: study design, country of study conduction, RD category, as defined in the literature,^4^ and specific RD. From this analysis emerged that only retrospective but not prospective studies documented significant differences in FtA concentrations between RD subjects and controls (SMD = −1.02; 95% CI −1.58 to −.46, *p* < .001; and −.14; 95% CI −1.93 to 1.65, *p* = .88, respectively, Figure S2), and that significant differences were reported in the countries were studies were conducted, except Turkey (Egypt SMD = −4.42; 95% CI −6.47 to −2.36, *p* < .001; Poland SMD = −.66; 95% CI −.85 to −.47, *p* < .001; Turkey SMD = .25; 95% CI −.16 to .66, *p* = .23; Figure S3), with no changes in heterogeneity.

**FIGURE 6:**
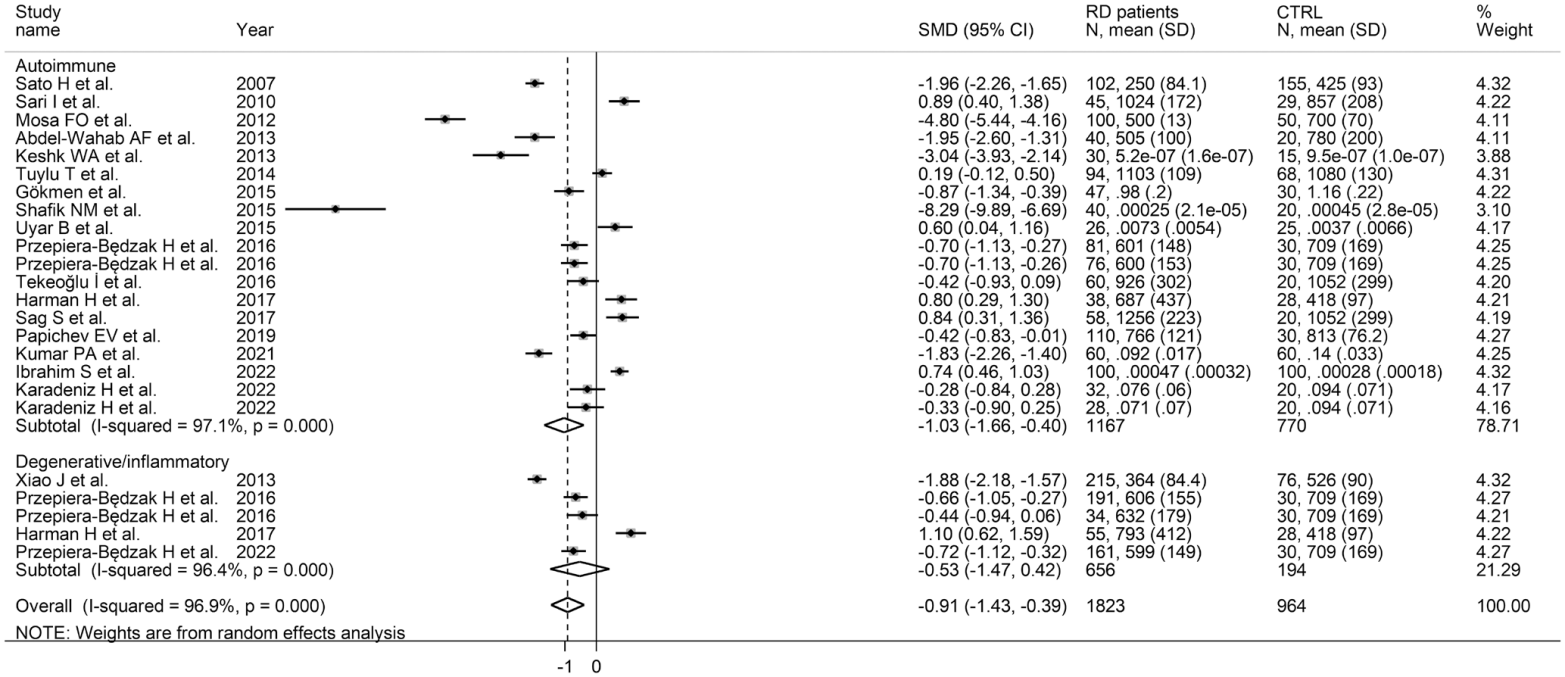
Forest plot of studies reporting FtA in autoimmune and degenerative/mixed RD.

**FIGURE 7:**
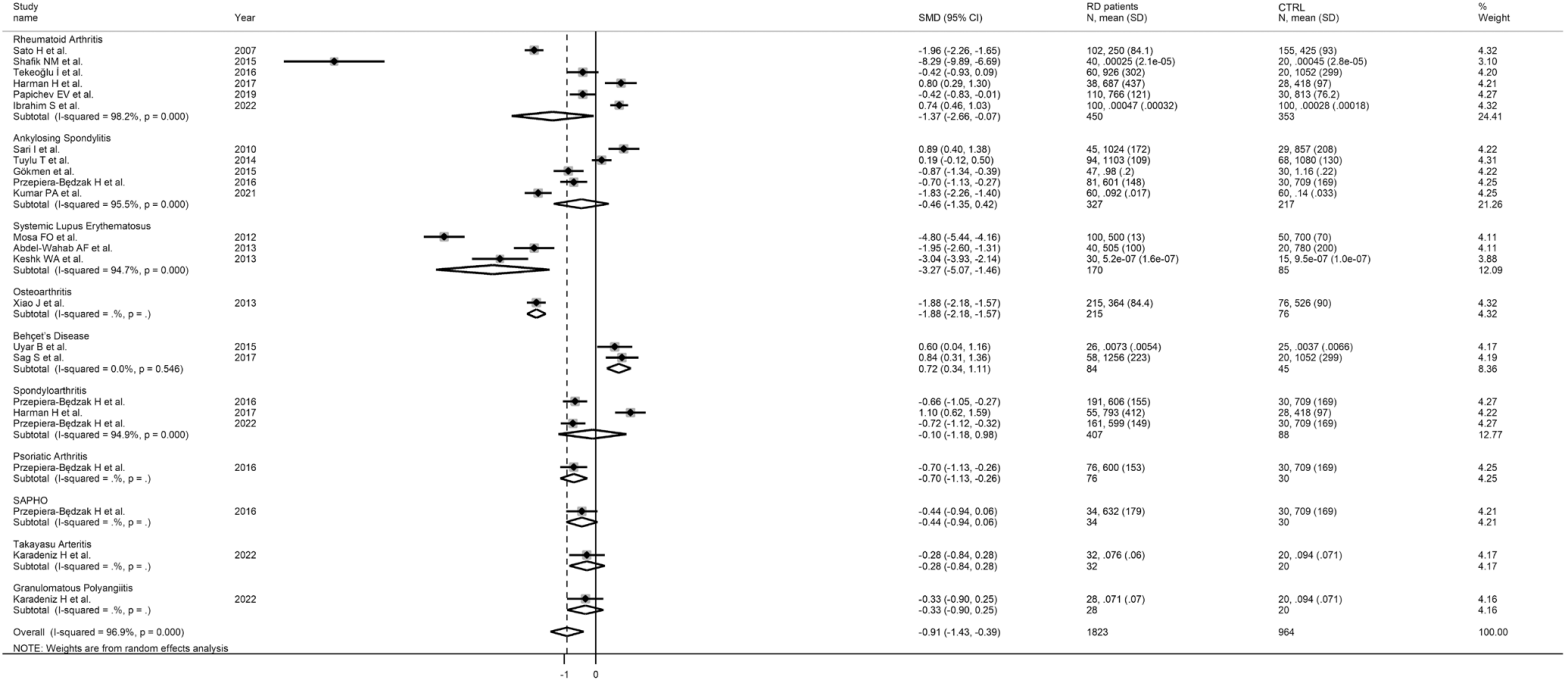
Forest plot of studies reporting FtA in RDs, stratified by the disease type.

Moreover, grouping the studies by their broad RD category highlighted that patients with autoimmune manifestations presented significantly decreased FtA concentrations compared to healthy subjects, while the difference was not significant in the group of degenerative/inflammatory RDs (SMD = −1.03, 95% CI −1.66 to −.40, *p* = .001; and SMD = −.53, 95% CI −.53 to .42, *p* = .27; Figure 6). Specifically, patients with RA and SLE had significantly lower FtA concentrations than controls (RA SMD = −1.37; 95% CI −2.66 to −.67, *p* = .04; SLE SMD = −3.27; 95% CI −5.07 to −1.47, *p* < .001); whereas no significant between‐group difference was reported in studies of patients with AS and SA (AS SMD = −.46; 95% CI −1.35 to .42, *p* = .31; SA SMD = −.10; 95% CI −1.18 to .98, *p* = .85); and an increase in FtA concentrations was reported in patients affected by BD (SMD = .72; 95% CI .34 to 1.11, *p* < .001). No changes in heterogeneity were observed (Figure 7).

#### 3.2.4 Certainty of evidence

The overall level of certainty was upgraded to moderate after considering the low‐moderate risk of bias in all studies (no change), the high but partially explainable heterogeneity (no change), the lack of indirectness (no change), the large effect size (SMD = −.91, upgrade one level) and the lack of publication bias (no change).

## 4 DISCUSSION

Our systematic review and meta‐analysis aimed at summarizing the state‐of‐the‐art evidence on hematic FtA concentrations in RD patients and healthy subjects, assessing possible associations with patients' characteristics, and stratifying the pooled effect size by study variables and specific RDs and RD categories. We were able to identify 19 published studies presenting data on RD patients in comparison with healthy subjects, finally including 24 RD cohorts of individuals with autoimmune, degenerative or autoinflammatory RD manifestation.

Our synthesis revealed a large negative effect size (SMD = −.91), demonstrating therefore that hematic FtA concentrations are significantly lower in RD patients. Moreover, no publication bias was found using the Begg's or the Egger's tests, and there was no need for correcting the pooled SMD through the application of the Duvall's ‘trim and fill’, therefore strengthening our findings.

To perform the univariate meta‐regression analysis, we calculated the ratio as RD patients and controls for each clinical and demographic parameter and assessed how it correlated with the observed effect size, finally highlighting a negative association between ESR ratio, total cholesterol ratio, LDL ratio and triglycerides ratio, and a positive association with higher HDL ratio in RD patients. Beside what already included in our systematic review and meta‐analysis, not many studies in the literature reported an association of FtA with ESR. Nevertheless, it has been demonstrated that FtA negatively correlated with ESR also in contexts different from RDs, like heart valve calcification^50^ and thoracic aortic aneurysms.^51^ Of note, this association was also found by Oncu et colleagues in the study on RD patients affected by familial Mediterranean fever (FMF).^52^ Although this study was not included in our systematic review because of the lack of a control group, this evidence supports our findings on RDs, reporting that FtA concentrations decreased in patients during the FMF episode compared to baseline values observed in the attack‐free period. On the contrary, FtA is a well‐known marker of cardiovascular disorders (CVD), with accumulating evidence suggesting that changes in FtA concentrations could raise the risk of death and morbidity from CVD.^53^ While low FtA concentrations were linked to a lower risk of CVD death in subjects with type 2 diabetes, and indicated also a higher risk of CVD mortality in older persons without the disease,^54^ others reported that individuals with high hematic FtA values were four times more likely than those with low levels to experience an ischemic stroke or a myocardial infarction.^55^ Furthermore, those who had previously experienced a myocardial infarction had noticeably greater FtA concentrations.^56^ Mori and colleagues^57^ hypothesized that FtA could play different roles in CVD accordingly to its concentration and to the stage of the disease, with low levels of serum FtA being linked to vascular calcification and high concentrations to insulin resistance and dyslipidaemia.^58^, ^59^, ^60^ Our synthesis revealed a negative association of FtA effect size with pro‐atherogenic factors of the lipid fraction (total cholesterol, LDL and triglycerides) and a positive association with the antiatherogenic factor HDL. Unfortunately, we did not collect enough evidence from the selected studies to further speculate on the prevalence of CVD in RD cohorts; however, only one of these studies reported an increased risk of endothelial dysfunction in patients with lower FtA levels.^47^

In the subgroup analysis, we did not observe significant changes in the heterogeneity of the included studies, although interesting aspects of our synthesis emerged. Indeed, our results indicated that only the cluster of retrospective studies significantly reported decreased blood FtA values in RD patients and that while hematic concentrations of FtA were decreased in all the geographic regions examined, there was an opposite trend in the Turkish region. Furthermore, while the group of RDs characterized by an autoimmune phenotype clearly reported a negative effect size, the autoinflammatory or degenerative RDs had a different trend. Focusing on the specific type of RD, we observed that patients with RA or SLE had reduced blood FtA concentrations, while, interestingly, subjects affected by BD had significantly higher values compared to healthy individuals, possibly due to different pathogenic mechanisms.

Finally, our systematic review and meta‐analysis highlighted numerous interesting features on FtA in RDs. Firstly, we contributed with a thorough assessment of the literature on hematic FtA concentrations in a wide range of RDs, analysing the outcome by RD type and phenotype, the study geographic distribution, and their methodology. We also assessed potential correlations between the observed measure of effect and the clinical and laboratory measurements, therapies and demographics of RD patients, although the biological plausibility of these associations needs to be addressed in further studies. The results were solid and not subjected to publication bias and were obtained in compliance with the PRISMA and GRADE guidelines. However, we believe that future efforts are needed to clarify some aspects that emerged from our synthesis: there is a clear need for more prospective studies and for additional evidence on specific RDs, like AS and SA, since the stratification by RD type highlighted specific influence of the characteristic traits of the disease on FtA concentration. Finally, although FtA can be easily monitored in serum, a limiting factor for its application is represented by the absence of accepted reference values: more research is required to determine if ethnicity could influence hematic FtA levels differently, requiring specific ranges in individual populations.

## 5 CONCLUSION

To conclude, we proved that FtA concentrations generally decrease in subjects affected by RDs although there are disorders like BD in which an opposite trend has been observed. The decrease also significantly correlated with increasing ESR values and with lipid profile variables. Nevertheless, further reports are needed to better understand the role of FtA in RD diseases and to determine whether this molecule could represent a good candidate for RD diagnosis, with the ultimate goal of improving patient care and life quality.

## AUTHOR CONTRIBUTIONS

B.D.L., S.Z and A.Z were involved in conceptualization. B.D.L., S.Z., A.A.M., P.P., G.L.E., C.C. and A.Z were involved in methodology. B.D.L., A.A.M. and A.Z. were involved in formal analysis. B.D.L., S.Z., A.A.M., P.P., G.L.E. and C.C. were involved in investigation and data curation. B.D.L., A.A.M. and A.Z. were involved in original draft preparation. B.D.L., S.Z., A.A.M., P.P., G.L.E., C.C. and A.Z. were involved in review and editing. C.C. and A.Z. were involved in supervision. All authors have read and agreed to the published version of the manuscript.

## FUNDING INFORMATION

No funding was received for conducting this study.

## CONFLICT OF INTEREST STATEMENT

The authors have no relevant financial or non‐financial interests to disclose.

## Supporting information


Appendix S1.

## Data Availability

Data supporting this study are included within the article and within the Appendix S1.
