# Supplementary material for: Circulating Fetuin‐A concentrations in rheumatic diseases: a systematic review and meta‐analysis
Source: Eur J Clin Invest. 2024 Nov 28;55(5):e14365. doi: 10.1111/eci.14365 (PMC12011677; doi:10.1111/eci.14365)

**Supplementary information**

**Complete search strategy**

**Pubmed**

("fetuin"[All Fields] OR “α2-Heremans-Schmid glycoprotein”) AND ("rheumatic diseases"[All Fields] OR "rheumatoid arthritis"[All Fields] OR "psoriatic arthritis"[All Fields] OR "reactive arthritis"[All Fields] OR "ankylosing spondylitis"[All Fields] OR "systemic lupus erythematosus"[All Fields] OR "systemic sclerosis"[All Fields] OR "scleroderma"[All Fields] OR "Sjogren's syndrome"[All Fields] OR "connective tissue diseases"[All Fields] OR "vasculitis"[All Fields] OR "Behcet's disease"[All Fields] OR "idiopathic inflammatory myositis"[All Fields] OR "polymyositis"[All Fields] OR "dermatomyositis"[All Fields] OR "gout"[All Fields] OR "pseudogout"[All Fields] OR "systemic vasculitis"[All Fields] OR "ANCA-associated vasculitis"[All Fields] OR "takayasu arteritis"[All Fields] OR "polyarteritis nodosa"[All Fields] OR "osteoarthritis"[All Fields] OR "fibromyalgia"[All Fields] OR "granulomatous polyangiitis"[All Fields] OR "henoch-schonlein purpura"[All Fields] OR "wegener granulomatosis"[All Fields])

**Web of Science**

(AB=(“fetuin” OR “α2-Heremans-Schmid glycoprotein” OR “α2-Heremans-Schmid glycoprotein”)) AND (AB=("rheumatic diseases" OR "rheumatoid arthritis" OR "psoriatic arthritis" OR " reactive arthritis" OR "ankylosing spondylitis" OR "systemic lupus erythematosus" OR "systemic sclerosis" OR " scleroderma" OR " Sjogren's syndrome " OR "connective tissue diseases" OR "vasculitis" OR "Behcet's disease" OR "idiopathic inflammatory myositis" OR "polymyositis" OR "dermatomyositis" OR "gout" OR "pseudogout" OR " systemic vasculitis" OR "ANCA-associated vasculitis" OR "takayasu arteritis" OR "polyarteritis nodosa" OR "osteoarthritis" OR "fibromyalgia"))

**SCOPUS**

TITLE-ABS-KEY ( "fetuin" OR “α2-Heremans-Schmid glycoprotein”) AND TITLE-ABS-KEY ( "rheumatic diseases" OR "rheumatoid arthritis" OR "psoriatic arthritis" OR " reactive arthritis" OR "ankylosing spondylitis" OR "systemic lupus erythematosus" OR "systemic sclerosis" OR " scleroderma" OR "Sjogren's syndrome" OR "connective tissue diseases" OR "vasculitis" OR "Behcet's disease" OR "idiopathic inflammatory myositis" OR "polymyositis" OR "dermatomyositis" OR "gout" OR "pseudogout" OR " systemic vasculitis" OR "ANCA-associated vasculitis" OR "takayasu arteritis" OR "polyarteritis nodosa" OR "osteoarthritis" OR "fibromyalgia" OR "granulomatous polyangiitis" OR " henoch-schonlein purpura" OR " wegener granulomatosis" )


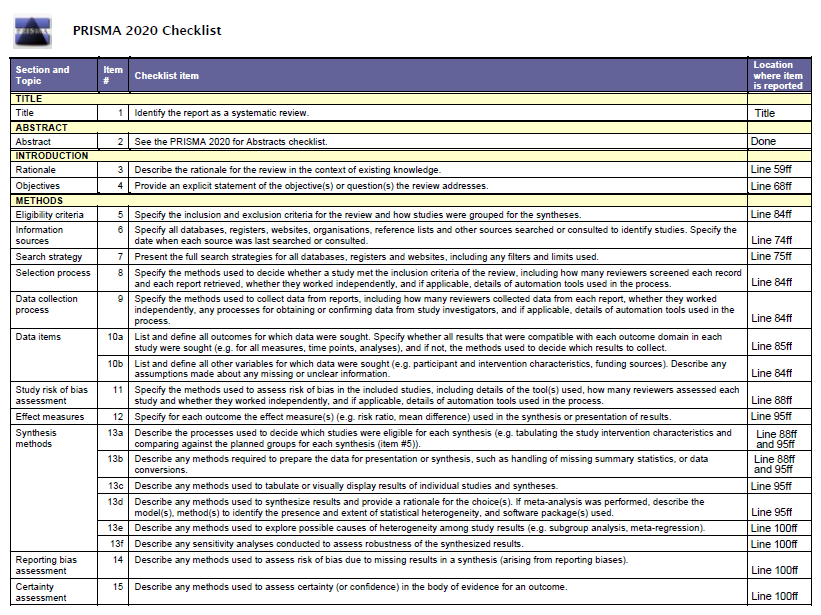

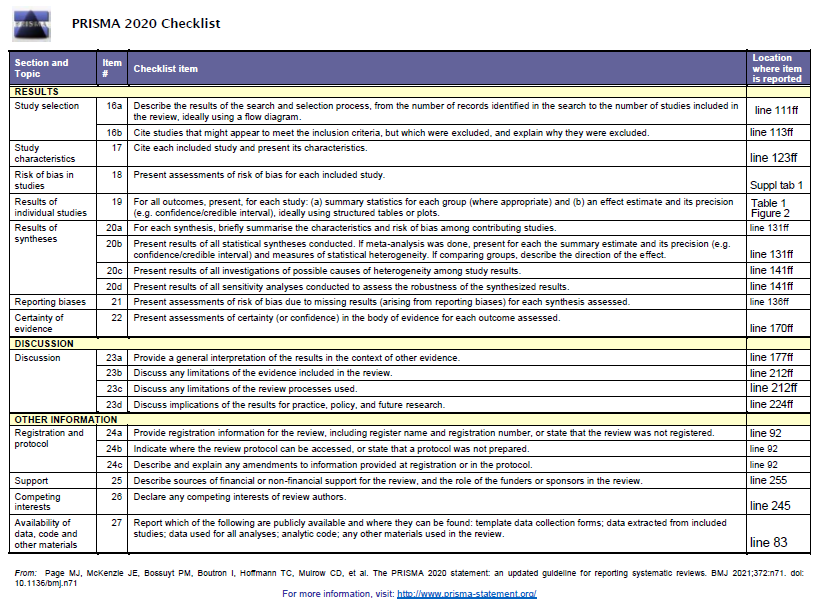


**Suppl. Table 1.** Assessment of the risk of bias using the Joanna Briggs Institute critical appraisal checklist.

| **Study** | **Were the inclusion criteria clearly defined?** | **Were the subjects and the setting described in detail?** | **Was the exposure measured in a reliable way?** | **Were standard criteria used to assess the condition?** | **Were confounding factors identified?** | **Were strategies to deal with confounding factors stated?** | **Were the outcomes measured in a reliable way?** | **Was appropriate statistical analysis used?** | **Risk of bias** |
| --- | --- | --- | --- | --- | --- | --- | --- | --- | --- |
| Sato H et al., 2007 | Yes | Yes | Yes | Yes | Yes | Yes | Yes | Yes | Low |
| Sari I et al., 2010 | Yes | Yes | Yes | Yes | Yes | Yes | Yes | Yes | Low |
| Mosa FO et al., 2012 | Yes | Yes | Yes | No | No | No | Yes | Yes | Moderate |
| Abdel-Wahab AF et al., 2013 | Yes | Yes | Yes | Yes | No | No | Yes | Yes | Low |
| Keshk WA et al., 2013 | Yes | Yes | Yes | Yes | No | No | Yes | Yes | Low |
| Xiao J et al., 2013 | Yes | Yes | Yes | Yes | Yes | Yes | Yes | Yes | Low |
| Tuylu T et al., 2014 | Yes | Yes | Yes | Yes | No | No | Yes | Yes | Low |
| Gökmen et al., 2015 | Yes | Yes | Yes | Yes | No | No | Yes | Yes | Low |
| Shafik NM et al., 2015 | Yes | Yes | Yes | Yes | Yes | Yes | Yes | Yes | Low |
| Uyar B et al., 2015 | Yes | Yes | Yes | Yes | No | No | Yes | Yes | Low |
| Przepiera-Będzak H et al., 2016 | No | Yes | Yes | Yes | Yes | Yes | Yes | Yes | Low |
| Tekeoğlu İ et al., 2016 | Yes | Yes | Yes | Yes | No | No | Yes | Yes | Low |
| Harman H et al., 2017 | Yes | Yes | Yes | Yes | No | No | Yes | Yes | Low |
| Sag S et al., 2017 | Yes | Yes | Yes | Yes | No | No | Yes | Yes | Low |
| Papichev EV et al., 2019 | No | Yes | Yes | Yes | No | No | Yes | Yes | Moderate |
| Kumar PA et al., 2021 | Yes | Yes | Yes | Yes | No | No | Yes | Yes | Low |
| Ibrahim S et al., 2022 | No | Yes | Yes | Yes | No | No | Yes | Yes | Moderate |
| Karadeniz H et al., 2022 | Yes | Yes | Yes | Yes | No | No | Yes | Yes | Low |
| Przepiera-Będzak H et al., 2022 | No | Yes | Yes | Yes | Yes | Yes | Yes | Yes | Low |

**Supplementary table 2. Summary of RD patients and CTRL clinical laboratory parameters, including:** C-reactive protein (CRP), erythrocyte sedimentation rate (ESR), total cholesterol (TChol), low and high density lipoprotein (LDL and HDL), and tryglicerides (TG).

| **Study name, Year** | **RD patients** | | | | | | | **CTRL** | | | | | |
| --- | --- | --- | --- | --- | --- | --- | --- | --- | --- | --- | --- | --- | --- |
|  | **Disease** | **CRP**  **mg/l** | **ESR**  **mm/h** | **TChol**  **mg/dl** | **LDL**  **mg/dl** | **HDL**  **mg/dl** | **TG**  **mg/dl** | **CRP**  **mg/l** | **ESR**  **mm/h** | **TChol**  **mg/dl** | **LDL**  **mg/dl** | **HDL**  **mg/dl** | **TG**  **mg/dl** |
| Sato H et al., 2007 | RA |  |  |  |  |  |  |  |  |  |  |  |  |
| Sari I et al., 2010 | AS | 9.1 | 11 | 173.7 | 108.2 | 45.9 | 95.7 | 2.5 | 3 | 184.9 | 112.7 | 49.8 | 92.3 |
| Mosa FO et al., 2012 | SLE |  |  | 154.5 | 106.5 | 27.7 | 127.3 |  |  | 154.3 | 93.7 | 42.7 | 73.6 |
| Abdel-Wahab AF et al., 2013 | SLE | 3.3 |  | 191.5 | 113 | 32.2 | 167.1 | 1.3 |  | 155.3 | 93.6 | 42.7 | 134.6 |
| Keshk WA et al., 2013 | SLE | 36.5 | 72 |  |  |  |  |  |  |  |  |  |  |
| Xiao J et al., 2013 | OA |  |  |  |  |  |  |  |  |  |  |  |  |
| Tuylu T et al., 2014 | AS | 9.8 |  |  |  |  |  | 3.3 |  |  |  |  |  |
| Gökmen et al., 2015 | AS | 11.7 | 30.7 |  |  |  |  | 2.8 | 10.6 |  |  |  |  |
| Shafik NM et al., 2015 | RA | 12.8 | 57.9 | 231.5 | 187 | 42.9 | 142.6 | 3.2 | 8.1 | 166.3 | 117.3 | 48.9 | 95.4 |
| Uyar B et al., 2015 | BD | 6.5 | 21.3 | 183.9 | 121.9 | 47.7 | 104.3 | 1.3 | 11.1 | 183.7 | 107.8 | 55 | 112.9 |
| Przepiera-Będzak H et al., 2016 | SpA | 6.6 | 15 | 209.8 | 127.7 | 59.5 |  |  |  | 229 | 139.2 | 62.5 |  |
|  | AS | 7.9 | 14.3 | 200 | 121.1 | 58.8 |  |  |  | 229 | 139.2 | 62.5 |  |
|  | PA | 5.3 | 13.7 | 217.4 | 132.1 | 59.4 |  |  |  | 229 | 139.2 | 62.5 |  |
|  | SAPHO | 6.1 | 19 | 218.1 | 135.1 | 61.6 |  |  |  | 229 | 139.2 | 62.5 |  |
| Tekeoğlu İ et al., 2016 | RA | 11.5 | 30.8 | 203.5 | 125.9 | 49 | 115.9 | 2.1 | 12.1 | 200.1 | 120.2 | 48.1 | 117.1 |
| Harman H et al., 2017 | SpA | 14.2 | 22.6 |  |  |  |  | 1.2 | 12.7 |  |  |  |  |
|  | RA | 13.6 | 27.8 |  |  |  |  | 1.2 | 12.7 |  |  |  |  |
| Sag S et al., 2017 | BD | 11.2 | 19.7 | 175.1 | 89.2 | 51.2 | 119.3 | 4.4 | 16.3 | 167 | 96.4 | 47.3 | 130.4 |
| Papichev EV et al., 2019 | RA |  |  |  |  |  |  |  |  |  |  |  |  |
| Kumar PA et al., 2021 | AS |  | 11.5 |  |  |  |  |  | 5.7 |  |  |  |  |
| Ibrahim S et al., 2022 | RA | 1.18 | 53.7 |  |  | 52.1 | 94 | 0.65 | 11.1 |  |  | 48.8 | 97 |
| Karadeniz H et al., 2022 | TA | 6 | 43 | 211.9 | 115.7 | 62.1 | 158.7 |  |  |  |  |  |  |
|  | GP | 12.9 | 53.2 | 192.9 | 111.3 | 50.8 | 138 |  |  |  |  |  |  |
| Przepiera-Będzak H et al., 2022 | SpA | 11.7 | 19.1 |  |  |  |  |  | 9 |  |  |  |  |

**Supplementary table 3. Summary of RD patients and CTRL clinical and demographical characteristics, including:** mean disease duration (MDD), body mass index (BMI), and percentage of individuals affected by hypertension (Hyp), diabetes mellitus (DM), and cardiovascular disorders (CVD).

| **Study name, Year** | **RD patients** | | | | | | | | **CTRL** | | | | | |
| --- | --- | --- | --- | --- | --- | --- | --- | --- | --- | --- | --- | --- | --- | --- |
|  | **Disease** | **MDD**  **(y)** | **Age** | **Males**  **%** | **BMI** | **Hyp**  **%** | **DM**  **%** | **CVD**  **%** | **Age** | **Males**  **%** | **BMI** | **Hyp**  **%** | **DM**  **%** | **CVD**  **%** |
| Sato H et al., 2007 | RA | 9.3 | 60.8 | 34.3 | 21.9 | 27.5 | 25.5 | 2 |  | 100 |  |  |  |  |
| Sari I et al., 2010 | AS | 13 | 37.4 | 77.8 | 25 |  |  |  | 35.5 | 82.2 | 24.8 |  |  |  |
| Mosa FO et al., 2012 | SLE |  |  |  |  |  |  |  |  |  |  |  |  |  |
| Abdel-Wahab AF et al., 2013 | SLE | 5.6 | 27.3 | 0 | 29.1 |  |  | 42.5 | 29.2 | 0 | 29.2 |  |  | 0 |
| Keshk WA et al., 2013 | SLE | 14.5 | 32.1 | 0 |  |  |  |  | 31.1 | 0 |  |  |  |  |
| Xiao J et al., 2013 | OA |  | 64.3 | 41.9 | 23.1 |  |  |  | 63.3 | 39.5 | 22.8 |  |  |  |
| Tuylu T et al., 2014 | AS | 15 | 42.2 | 69.1 |  | 4.3 | 2.1 |  | 44.2 | 70.6 |  | 1.5 | 0 |  |
| Gökmen et al., 2015 | AS | 5.4 | 39.7 | 63.8 | 27.3 |  |  |  | 42 | 53.3 | 28.8 |  |  |  |
| Shafik NM et al., 2015 | RA | 9.7 | 38.7 | 0 | 23 |  |  |  | 40.8 | 0 | 24.2 |  |  |  |
| Uyar B et al., 2015 | BD | 7.1 | 35.1 | 39.1 | 27 |  |  |  | 32.3 | 48 | 25.9 |  |  |  |
| Przepiera-Będzak H et al., 2016 | SpA | 5.7 | 48.3 | 50.8 | 26.4 | 24.6 | 31.4 | 9.9 | 43.5 | 36.7 | 24.2 |  |  |  |
|  | AS | 10 | 44.7 | 75.3 | 26 | 30.9 | 2.5 | 12.3 | 43.5 | 36.7 | 24.2 |  |  |  |
|  | PA | 4.7 | 50.8 | 43.4 | 26.9 | 17.1 | 1.3 | 9.2 | 43.5 | 36.7 | 24.2 |  |  |  |
|  | SAPHO | 2.7 | 51.7 | 8.8 | 26.8 | 26.5 | 8.8 | 5.9 | 43.5 | 36.7 | 24.2 |  |  |  |
| Tekeoğlu İ et al., 2016 | RA |  | 53.5 | 20 |  |  |  |  | 49.4 | 25 |  |  |  |  |
| Harman H et al., 2017 | SpA | 20.8 | 38.8 | 72.7 | 26.2 |  |  |  | 38.8 | 35.7 |  |  |  |  |
|  | RA | 9.1 | 45.3 | 26.3 | 26.2 |  |  |  | 38.8 | 35.7 |  |  |  |  |
| Sag S et al., 2017 | BD |  | 38.3 | 38 |  |  |  |  | 39.8 | 25 |  |  |  |  |
| Papichev EV et al., 2019 | RA |  |  | 100 |  |  |  |  |  | 100 |  |  |  |  |
| Kumar PA et al., 2021 | AS | 5.9 | 38.5 | 100 | 25.3 |  |  |  |  | 100 | 24.3 |  |  |  |
| Ibrahim S et al., 2022 | RA |  | 46.4 | 55 |  |  |  |  | 47.8 | 64 |  |  |  |  |
| Karadeniz H et al., 2022 | TA |  | 36.4 | 3.1 |  |  |  |  | 41.3 | 55 |  |  |  |  |
|  | GP |  | 51.6 | 57.1 |  |  |  |  | 41.3 | 55 |  |  |  |  |
| Przepiera-Będzak H et al., 2022 | SpA | 11.9 | 46.2 | 76.4 |  | 23.6 |  | 8.1 | 43.5 | 100 |  |  |  |  |

**Suppl. Figure 1.** Funnel plot of inverse of the variance plotted against the FtA effect size.


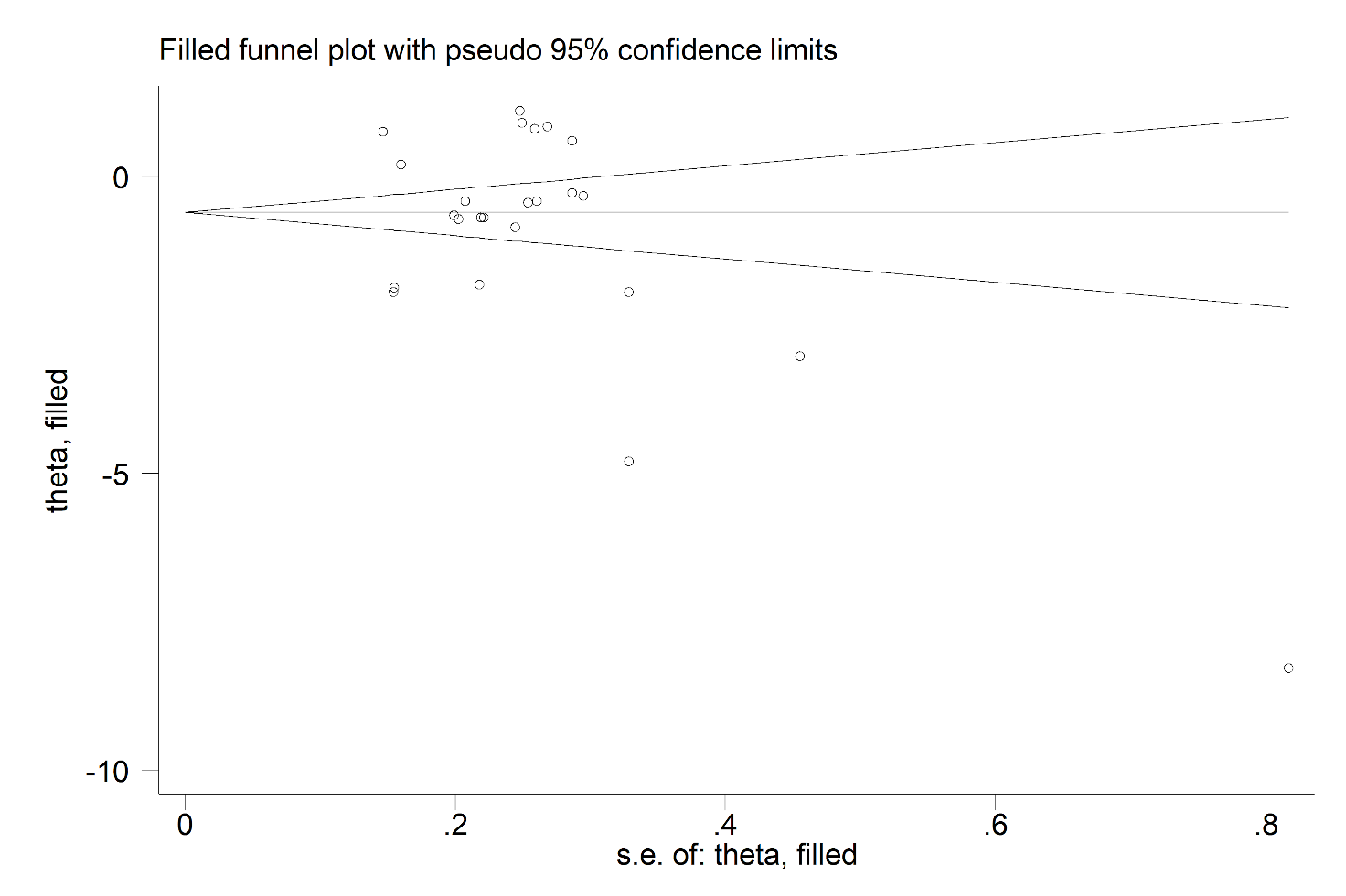


**Suppl. Figure 2.** Forest plot of studies stratified by study design.


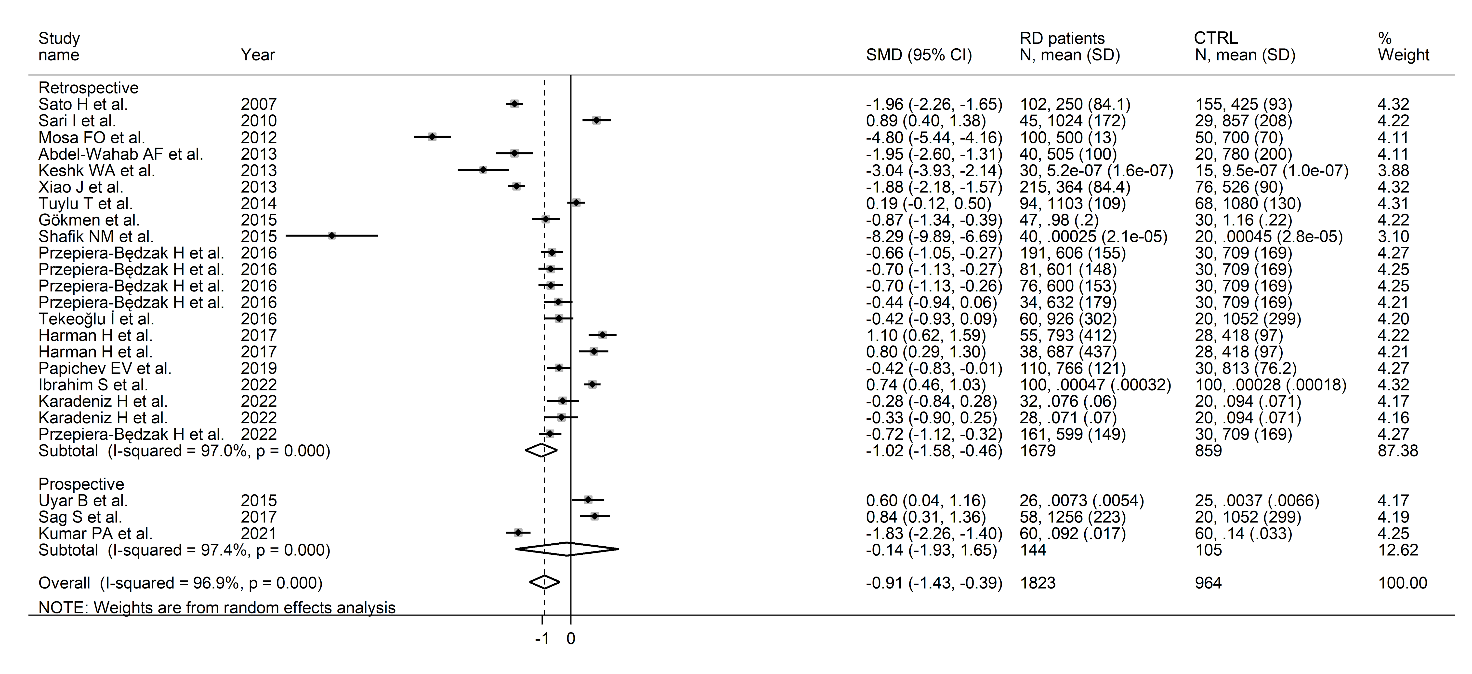


**Suppl. Figure 3.** Forest plot of studies stratified by country of study conduction.


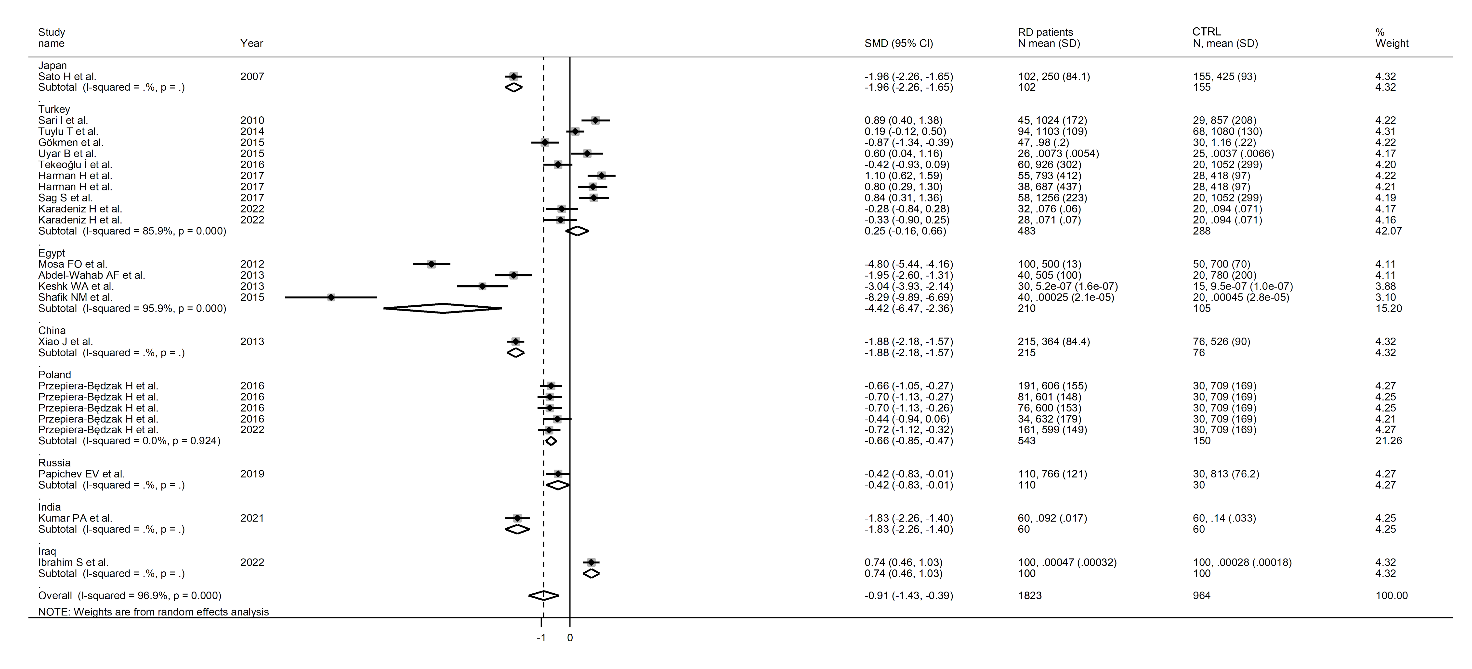

Supplement: Supplementary file 1 — Appendix S1. [file ECI-55-e14365-s001.docx]
